# Intensity-modulated stereotactic body radiotherapy for stage I non-small cell lung cancer

**DOI:** 10.3892/ol.2012.1082

**Published:** 2012-12-18

**Authors:** MIN-JEONG KIM, SEUNG-GU YEO, EUN SEOK KIM, CHUL KEE MIN, PYUNG SE AN

**Affiliations:** 1 Department of Radiology, Hallym Sacred Heart Hospital, Hallym University College of Medicine, Anyang;; 2 Department of Radiation Oncolocy, Soonchunhyang University Hospital; Cheonan, Republic of Korea; 3 Department of Radiation Oncology, Soonchunhyang University College of Medicine, Cheonan, Republic of Korea

**Keywords:** non-small cell lung cancer, medically inoperable, stage I, stereotactic body radiotherapy, intensity-modulated radiotherapy

## Abstract

This study aimed to investigate the clinical outcomes of intensity-modulated radiotherapy (IMRT)-based stereotactic body radiotherapy (SBRT) for patients with stage I non-small cell lung cancer (NSCLC). A prospective database of 16 consecutive patients receiving SBRT for pathologically-proven and peripherally-located stage I NSCLC was reviewed. Fifteen patients were medically inoperable and one patient refused to undergo surgery. The median age of the patients was 76 years (range, 69–86). Treatment planning used four-dimensional computed tomography and fixed-field IMRT (n=11) or volumetric-modulated arc therapy (VMAT; n=5). The SBRT scheme was 48 Gy in four fractions (n=9) or 55 Gy in five fractions (n=7), delivered on consecutive days. The overall response rate at 6 months was 78.6%, including a complete response in three (21.4%) patients and a partial response in eight (57.1%). Three patients (21.4%) demonstrated a stable disease status. The median follow-up time was 14 months (range, 6–20) for the surviving patients. One patient developed local failure at 11 months, while another suffered from regional failure in a subcarinal lymph node at 4 months. Two patients did not survive within the first 6 months; one patient died during salvage chemotherapy for mediastinal lymph node metastasis and the other succumbed to a cause unrelated to lung cancer. The Kaplan-Meier estimates of local failure-free, progression-free and overall survival rates at 18 months were 91.0, 85.2 and 87.5%, respectively. The toxicity was mild; no severe (grade ≥3) toxicity was identified. IMRT-based (including VMAT) delivery of SBRT for patients with stage I NSCLC demonstrated favorable responses and local control without severe toxicity.

## Introduction

Anatomical resection is the standard treatment for early-stage lung cancer, yielding a locoregional control rate of ∼90% and a 5-year overall survival rate of 50–70% for stage I non-small cell lung cancer (NSCLC) (1). However, a significant proportion of NSCLC patients present with comorbidities and an advanced age, causing them to be deemed medically inoperable. Chronic obstructive pulmonary disease with emphysema and pronounced reduction of lung capacity accounts for the majority of inoperable patients (2). Moreover, certain patients are unwilling to undergo surgery. These patients are primarily referred for radiation therapy (RT); with conventional RT, the rate of local control has historically been poor (30–70%), with an overall 5-year survival rate of only 15–30% (3,4). Otherwise, patients refuse RT due to the long treatment period and are observed without specific cancer therapy. The reason for poor tumor control with conventional RT has been revealed to be an insufficient total radiation dose, which is typically ≤60 Gy (4).

Stereotactic body RT (SBRT), also referred to as stereotactic ablative RT, is a form of high-precision RT for tumor targets in extracranial sites, employing higher doses per fraction and fewer fractions than conventional RT (5). SBRT delivers a much higher biological effective dose (BED) compared with conventional RT and has reduced local failure (<10%) comparable to the rates following surgery, in patients with early-stage NSCLC (2,6–10).

In SBRT for lung cancer, a basic principle of RT, to maximize the dose of radiation delivered to a tumor and to spare normal tissue, becomes even more important. This is due to the fact that rather than the differential radiosensitivities of normal and target tissues, the geometry and/or intensity of the beams is the predominant factor in sparing normal tissues (5). Studies have suggested that the use of intensity-modulated RT (IMRT) in the process of radiosurgery or SBRT has the potential to improve tumor coverage and spare normal tissue (11,12). Volumetric-modulated arc therapy (VMAT) is a novel extension of IMRT, reducing treatment times by radiation delivery in a gantry rotation up to 360° with a dynamic multi-leaf collimator motion, variable dose rates and gantry speed modulation (13). However, few clinical studies have been conducted that have adopted IMRT/VMAT during SBRT for lung cancer.

SBRT is particularly challenging due to the added complexities introduced by target motion during natural physiological processes, such as respiration. Four-dimensional (4D) computed tomography (CT) scans that correlate CT images with respiratory phases have frequently been employed to take into account respiration-related tumor motion (14). Integrated imaging devices in treatment units presently allow CT scans (cone-beam CT) to be performed immediately prior to treatment, while the patient is on the treatment couch, thereby confirming that the patient and tumor are positioned correctly (15).

In the present study, we report our clinical investigation of SBRT in patients with stage I NSCLC. 4D CT imaging, IMRT/VMAT for planning/delivery and image-guided RT with cone-beam CT were employed. The clinical outcomes, including treatment response rate, local disease control rate and toxicity, were analyzed.

## Patients and methods

### Patients

Between December 2010 and March 2012, a total of 16 consecutive patients with primary NSCLC were treated using SBRT. Patient- and treatment-related data were collected from a prospectively registered database. Inclusion criteria for SBRT were as follows: Pathologically confirmed NSCLC; clinical stage T1-2N0M0 according to the American Joint Committee on Cancer Staging Manual, 7th edition (16); longest tumor diameter <5 cm; and Eastern Cooperative Oncology Group performance scale score ≤2. Only patients who were considered to be inoperable due to poor medical condition or refusal to undergo surgery were included. The treated tumor was required to be further than 2 cm in all directions from the proximal bronchial tree, which was defined as the distal 2 cm of the trachea, carina and major lobar bronchi up to their first bifurcation (7). Patients who had previously undergone chemotherapy or RT for lung cancer were excluded. All patients provided written informed consent, and the study was conducted in compliance with the Declaration of Helsinki (17). The study was approved by the ethics committee of Soonchunhyang University Hospital (Cheonan, Korea).

Before initiation of treatment, a complete history was taken and patients underwent a physical examination, contrast-enhanced CT imaging of the chest, ^18^F-fluorodeoxyglucose positron emission tomography (PET)-CT scanning, a pulmonary function test and brain imaging (contrast-enhanced CT or magnetic resonance imaging).

### Treatment

The patients were treated using a Novalis Tx system (Varian Medical Systems, Palo Alto, CA, USA and BrainLab, Feldkirchen, Germany). During the simulation, patients were immobilized in the supine position, with the arms above the head, in a vacuum-bag restriction system (Vac-Lock, Civco Medical Solutions, Kalona, IA, USA). Respiration-correlated 4D CT scans were performed during uncoached quiet respiration using a Real-Time Position Management (RPM) system (Varian Medical Systems) and a 16-slice CT scanner (Brilliance CT Big Bore, Philips Medical Systems, Cleveland, OH, USA). Data were acquired for the duration of a full respiratory cycle. Each reconstructed image was assigned to a specific respiratory phase to collectively yield a set of 10 CT images, each of which reflected 10% of the respiratory cycle. The gross tumor volume was delineated on the CT image for each respiratory phase using the ‘lung window’ setting. No expansion was made to account for microscopic disease extent, and the clinical target volume was equivalent to the gross tumor volume. To encompass the entire trajectory of the target, an internal target volume was generated from the sum of the gross tumor volumes during all 10 respiratory phases. The planning target volume (PTV) was created by adding a 0.5-cm isotropic set-up margin around the internal target volume. Critical structures, including the lungs, spinal cord, esophagus, trachea, proximal bronchial tree, heart, great vessels, ribs and skin, were outlined. Normal tissue dose volume constraints were adapted from data in the Radiation Therapy Oncology Group SBRT trial protocols (18).

All plans were created using the Eclipse treatment planning system (Varian Medical Systems) and 6-MV photons, taking into account inhomogeneity corrections. A fixed-field IMRT plan was generated using 7–9 non-opposing coplanar beams. In the VMAT plan, 2–4 partial arcs were used. The same optimization objectives and penalties were used for the IMRT and VMAT plans. The dose fractionation schedules were 48 Gy/4 fractions or 55 Gy/5 fractions, delivered on consecutive days. Dosimetric criteria mandated that 95% of the PTV was covered conformally by the prescription dose and that 99% of the PTV received 90% of the prescription dose. The cone-beam CT images of the tumor were registered to the contours and images from the 4D CT planning data sets and were used to guide patient localization. Pre-treatment cone-beam CT and patient repositioning were repeated when the set-up error was estimated to be ≥3 mm in any direction.

### Evaluation and analysis

Patients were followed up every 3 months during the first and second years, and every 6 months thereafter. Follow-up CT scans were performed at each visit, but PET-CT scans were repeated only in the event of suspected disease relapse. Tumor measurements at each follow-up appointment were performed using the Response Evaluation Criteria in Solid Tumors (19), in which a complete response (CR) is total tumor disappearance and a partial response (PR) is a decrease of ≥30% in the longest tumor diameter. Local control and survival were measured from the time of diagnosis. Local failure was defined as progressive and increasing CT scan abnormalities that were confirmed by progressive and incremental increases in the standardized uptake values of a lesion in serial PET-CT imaging, with or without biopsy. Tumor progression in the hilar, mediastinal or supraclavicular lymph nodes was considered regional failure. The National Cancer Institute’s Common Toxicity Criteria (version 3.0) were used to grade adverse events. Survival was estimated using the Kaplan-Meier method and statistical analyses were performed using the Statistical Package for the Social Sciences (SPSS) software, version 14.0 (SPSS, Chicago, IL, USA).

## Results

### Patients

The patient, tumor and treatment characteristics are summarized in Table I. The median patient age was 76 years (range, 69–86) and 12 (75%) patients were male. Nine of the patients’ tumors were clinically staged as T1N0M0, while seven were T2N0M0. The histological subtypes were squamous cell carcinoma in nine patients, adenocarcinoma in six and large cell neuroendocrine carcinoma in one. The maximal tumor diameter ranged from 1.5 to 5 cm (median, 2.8). Fifteen patients were not appropriate candidates for surgery due to chronic pulmonary disease, poor lung function, advanced age or other chronic illnesses, and one patient refused to undergo surgery. Two patients had a history of NSCLC or rectal cancer, diagnosed eight and 10 years, respectively, prior to the current presentation. In one patient with previous NSCLC (squamous), pneumonectomy of the right lung was performed and novel NSCLC (squamous) developed in the left lung. Conventional planning CT, as opposed to 4D CT, was performed in one patient who suffered from severe kyphosis and required treatment in a prone position.

### Response and local control

**Figure 1:**
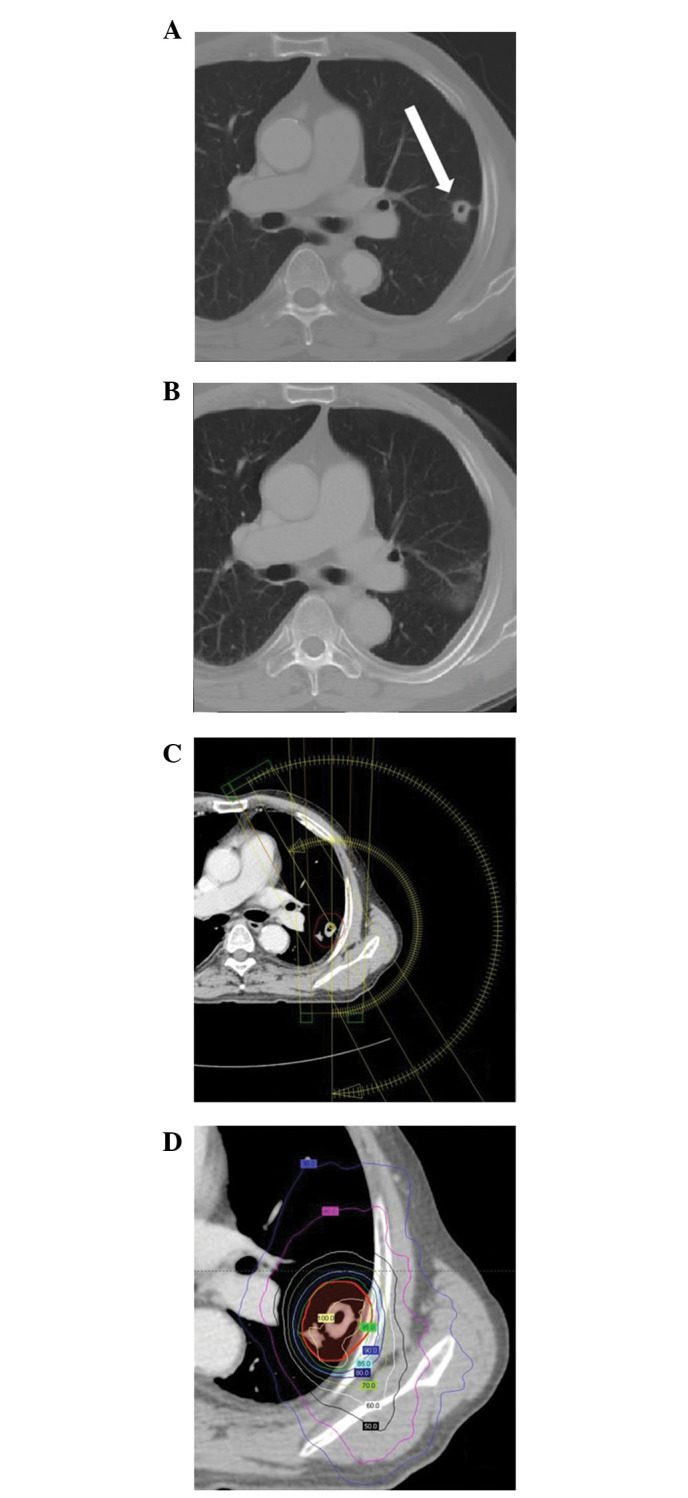
Radiographic change and treatment plan of a patient who achieved a complete response. (A) Chest computed tomography (CT) scan prior to treatment, revealing a 1.5-cm cavitary lesion (white arrow) in the left upper lobe (cT1aN0, squamous cell carcinoma). (B) Chest CT scan at 6 months, revealing no definitive lesion with a band-like opacity representing the radiation-induced change. (C) Treatment planning was conducted using volumetric-modulated arc therapy (2 partial arcs). The fractionation scheme was 55 Gy/5 fractions. (D) Dose distribution with isodose lines of different colors. The planning target volume is fully enclosed by a 95% isodose line.

The median follow-up period was 13 months (range, 4–20) for all patients and 14 months (range, 6–20) for surviving patients. In the 14 evaluable patients, the response rate at 6 months, consisting of all patients with a CR (n=3; 21.4%) or a PR (n=8; 57.1%), was 78.6% (11/14). A typical case of a CR is presented in Fig. 1. The remaining three patients (21.4%) achieved a stable disease status. Two non-evaluable patients, who did not survive the first 6 months, demonstrated stable disease at 3 months.

One patient developed local failure 11 months after SBRT. Another patient demonstrated regional failure in a subcarinal lymph node at 4 months. All relapses were confirmed by a combination of CT and PET-CT. Two patients did not survive; one of whom developed subcarinal lymph node metastasis and died during salvage chemotherapy at 5 months, while the other succumbed to a cause unrelated to lung cancer (a cardiopulmonary event) at 4 months. The Kaplan-Meier estimates of local failure-free, progression-free and overall survival rates at 18 months were 91.0, 85.2 and 87.5%, respectively.

### Toxicity

All patients completed SBRT with no treatment interruption. Despite the medical comorbidities and advanced age of the patients, SBRT was well-tolerated. Five patients (31.3%) reported no toxicities. Grade 1 toxicities included pulmonary toxicity in seven patients (43.8%), transient mild erythema in three patients (18.8%), fatigue in two patients (12.5%) and dysphagia in one patient (6.3%). Grade 2 toxicities included pulmonary toxicity in four patients (25.0%) and chest pain in two patients (12.5%). No toxicity ≥ grade 3 was observed.

## Discussion

The present study analyzed patients with stage I NSCLC receiving SBRT, in whom the 6-month response rate was 78.6% (CR, 21.4%; PR, 57.1%). Response rates for lung SBRT have been described by a number of authors, and treatment response rates following SBRT for lung cancer have been found to improve until ∼1 year post-treatment (7–10,20). Timmerman *et al* reported CR and PR rates of 51% and 38%, respectively, following SBRT in 55 patients with early-stage NSCLC (7). A CR occurred at 1.6 –42.6 months (median, 6.5) after the completion of SBRT. Mohammed *et al* investigated the time course of radiographic tumor responses following SBRT for primary or metastatic lung tumors (20). The CR and PR rates were 3 and 43% at 6 weeks, 15 and 38% at 4 months, and 27 and 64% at 1 year, respectively. Taremi *et al* analyzed 108 patients with stage I NSCLC receiving SBRT and the treatment response rate was greater at 1 year (CR, 30.5%; PR, 37.5%) compared with at 3 months (CR, 7%; PR, 68.4%) (9). Further follow-up of patients in the present study may reveal greater responses than those described here.

Local control and survival rates at 18 months (91.0 and 87.5%, respectively) were comparable to previously demonstrated outcomes (2,6–10). Disease relapse occurred in two patients and one patient showed mediastinal lymph node metastasis at 4 months. As the metastasis appeared soon after SBRT, we speculate that the patient harbored occult tumors at diagnosis that went undetected during initial CT and PET staging. Local disease progression developed in another patient after 11 months. Local failure may be due to either a geographic miss or radiation resistance, even with high BEDs. The former appeared to have a greater influence as a growing mass appeared within 1 cm of the PTV. Notably, the patient’s planning process did not involve 4D CT due to severe kyphosis.

The 4D CT scans provide information on not only the extent of tumor motion but also the different spatial tumor positions (14). A target volume that includes only areas with demonstrable tumor appearance is smaller than the conventional PTV, which includes greater non-specific and universal isotropic margins and covers areas that do not harbor the tumor at any point during the respiration cycle. In addition to this smaller volume, 4D-based target definition is important in avoiding target misses (21). Normal treatment planning CT using modern CT scanners that acquire scans in a short time (fast-CT) only displays the tumor for a certain moment of the respiration cycle and may contain motion-induced artifacts that cause inadequate visualization of the tumor (22). Underberg *et al* evaluated the differences between 4D CT-based target volumes and targets defined in several consecutive fast-CT scans; the 4D scans captured motion that was missed by fast-CT (23). The present study supports the use of 4D CT in SBRT planning for lung lesions.

Non-clinical planning studies have validated the suitability of IMRT for the setting of radiosurgery or SBRT. These studies have demonstrated significant dosimetric improvements for small and irregularly shaped lesions compared with the results of other techniques, with reductions in critical organ irradiation (11,24). However, few published clinical studies have adopted IMRT during SBRT for lung cancer (12). Although we did not compare dosimetric parameters with those from conventional three-dimensional planning in the present study, highly conformal target coverage with homogenous dose distribution, and with radiation exposure of normal tissue well below the recommended dose volume constraints, was achieved with IMRT. None of the patients, including the patient with previous pneumonectomy, experienced severe (≥ grade 3) toxicity. In addition to the radiation dose distribution, treatment time also requires consideration in SBRT planning/delivery, as SBRT for lung tumors is mostly applied in medically inoperable patients who are often elderly with other medical problems. Decreases in the treatment time associated with VMAT are capable of reducing the likelihood of patient movement as a result of discomfort and minimizing the random error introduced by intrafraction tumor motion (13,25). Five patients in the present study, who were more fragile, were treated using VMAT, which permitted a reduction in the beam-on time. If dosimetric parameters are not inferior to those for fixed-field IMRT, we plan to preferentially treat patients using this technique. More detailed descriptions of the IMRT/VMAT methods and dosimetric analysis will be presented in a further study.

Two fractionation schedules, 48 Gy/4 fractions and 55 Gy/5 fractions, were implemented in the present study. BEDs calculated using a linear-quadratic model (α/β assumed to be 10) were 105.6 and 115.5 Gy_10_, respectively (26). These BEDs have been more widely adopted in Japan and are lower than those with the 60 Gy/3 fraction scheme (BED=180 Gy_10_) mainly employed in North America (6,10). A BED >100 Gy_10_ is generally accepted as an adequate cut-off dose; below this threshold, local failure risk is higher (10,27). However, Stephans *et al* demonstrated no difference in local control or survival rates between 50 Gy/5 fractions and 60 Gy/3 fractions in SBRT for patients with medically inoperable stage I NSCLC, and chest wall toxicity was more common with the latter scheme (28). When the one episode of local recurrence is regarded as being due to a geographical miss, the 100% local control rate in the present study suggests that there may be no large dose-response gain above these modest BEDs. However, the present study requires further follow-up; the optimum dose fractionation in SBRT for lung cancer may be elucidated through prospective randomized studies.

In conclusion, the current study provides additive evidence for establishing the favorable efficacy and safety of SBRT for patients with stage I NSCLC. Novel techniques using IMRT/VMAT were feasible in lung SBRT, and 4D CT was demonstrated to be necessary for simulation and planning in order to precisely account for tumor motion during respiration.

## Figures and Tables

**Table I:** Patient, tumor and treatment characteristics.

Characteristic	No.
Age (years)	
Median	76
Range	69–86
Gender	
Male	12
Female	4
Pathology	
Squamous	9
Adenocarcinoma	6
Large cell neuroendocrine	1
cT classification	
cT1a	4
cT1b	5
cT2a	7
Tumor size (cm)	
Median	2.8
Range	1.5–5.0
Tumor location (lobe)	
Left upper/lower	6/3
Right upper/middle/lower	2/1/4
PTV volume (ml)	
Median	89.3
Range	43.4–223.5
Fractionation scheme	
48 Gy/4 fractions	9
55 Gy/5 fractions	7
SBRT technique	
Fixed-field IMRT	11
VMAT	5

PTV, planning target volume; SBRT, stereotactic body radiotherapy; IMRT, intensity-modulated radiotherapy; VMAT, volumetric-modulated arc therapy.
